# Validity of the modified Rankin Scale in patients with aneurysmal subarachnoid hemorrhage: a randomized study

**DOI:** 10.1186/s12883-023-03479-x

**Published:** 2024-01-12

**Authors:** E. Nobels-Janssen, E. N. Postma, I. L. Abma, J. M. C. van Dijk, I. R. de Ridder, H. Schenck, W. A. Moojen, M. H. den Hertog, D. Nanda, A. R. E. Potgieser, B. A. Coert, W. I. M. Verhagen, R. H. M. A. Bartels, P. J. van der Wees, D. Verbaan, H. D. Boogaarts

**Affiliations:** 1 Department of Neurology, Haga Teaching Hospital, The Hague, The Netherlands; 2 Department of Neurosurgery, Radboud University Medical Center, PO Box 9101, Nijmegen, 6500 HB The Netherlands; 3 Amsterdam UMC, Department of Neurosurgery, Amsterdam Neuroscience, University of Amsterdam, Amsterdam, The Netherlands; 4 IQ healthcare and Department of Rehabilitation, Radboud University Medical Center, Radboud Institute of Health Sciences, Nijmegen, The Netherlands; 5 Department of Neurosurgery, University Medical Center Groningen, Groningen, The Netherlands; 6 Department of Neurology, Maastricht University Medical Center, Cardiovascular Research Institute, Maastricht, The Netherlands; 7 Department of Neurosurgery, Haaglanden Medical Center, The Hague, The Netherlands; 8 Department of Neurosurgery, Leiden University Medical Center, Leiden, The Netherlands; 9 Department of Neurosurgery, Haga Teaching Hospital, The Hague, The Netherlands; 10 Department of Neurology, Isala Hospital, Zwolle, The Netherlands; 11 Department of Neurosurgery, Isala Hospital, Zwolle, The Netherlands; 12 Department of Neurosurgery, Medisch Spectrum Twente, Enschede, The Netherlands

**Keywords:** Modified Rankin Scale, Convergent validity, Responsiveness, Subarachnoid hemorrhage

## Abstract

**Purpose:**

The modified Rankin Scale (mRS), a clinician-reported outcome measure of global disability, has never been validated in patients with aneurysmal subarachnoid hemorrhage (aSAH). The aims of this study are to assess: (1) convergent validity of the mRS; (2) responsiveness of the mRS; and (3) the distribution of mRS scores across patient-reported outcome measures (PROMs).

**Methods:**

This is a prospective randomized multicenter study. The mRS was scored by a physician for all patients, and subsequently by structured interview for half of the patients and by self-assessment for the other half. All patients completed EuroQoL 5D-5L, RAND-36, Stroke Specific Quality of Life scale (SS-QoL) and Global Perceived Effect (GPE) questionnaires. Convergent validity and responsiveness were assessed by testing hypotheses.

**Results:**

In total, 149 patients with aSAH were included for analysis. The correlation of the mRS with EQ-5D-5L was r = − 0.546, while with RAND-36 physical and mental component scores the correlation was r = − 0.439and r = − 0.574 respectively, and with SS-QoL it was r = − 0.671. Three out of four hypotheses for convergent validity were met. The mRS assessed through structured interviews was more highly correlated with the mental component score than with the physical component score of RAND-36. Improvement in terms of GPE was indicated by 83% of patients; the mean change score of these patients on the mRS was − 0.08 (SD 0.915). None of the hypotheses for responsiveness were met.

**Conclusion:**

The results show that the mRS generally correlates with other instruments, as expected, but it lacks responsiveness. A structured interview of the mRS is best for detecting disabling neuropsychological complaints.

**Registration:**

URL: https://trialsearch.who.int; Unique identifier: NL7859, Date of first administration: 08-07-2019

**Supplementary Information:**

The online version contains supplementary material available at 10.1186/s12883-023-03479-x.

## Introduction

**Fig. 1:**
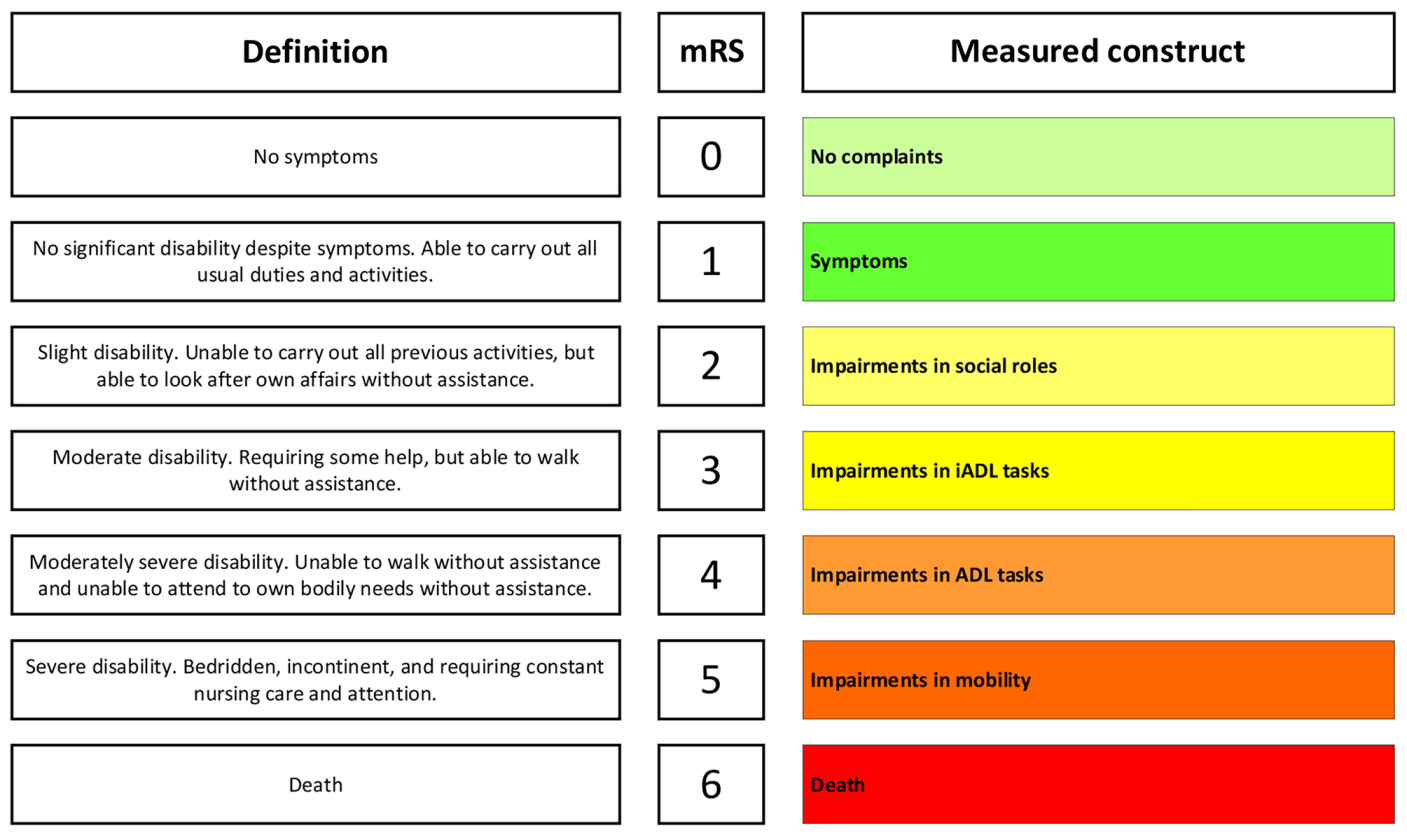
Overview of the modified Rankin Scale (mRS) illustrating the definitions and different constructs of which the mRS is composed. In the left column the established definition as formulated by Rankin is illustrated[2]; in the right column we illustrate the construct that is measured for that specific mRS score. This figure shows that if there is an impairment in a certain domain, the mRS score will be at least the corresponding number or higher Abbreviations: mRS: ADL: activities of daily living; iADL: instrumental activities of daily living; modified Rankin Scale

The Rankin scale was developed in 1957 to assess outcomes in stroke patients and modified in the 1980s to improve its comprehensiveness [1]. The modified Rankin scale (mRS) is an ordinal seven-point scale ranging from no residual symptoms to severely disabled and death (Fig. 1) [2]. It is a clinician-reported measure of global disability or, more precisely, mobility and disability in basic and instrumental activities of daily living ((I)ADL). The measured construct depends on the value of the mRS score (Fig. 1). The mRS is one of the most frequently used outcome measures in randomized clinical trials in patients with aneurysmal subarachnoid hemorrhage (aSAH) [3], but the mRS has never been validated in this population, who often display fewer physical handicaps than those with ischemic stroke [4].

Approximately half of the patients who survive aSAH experience problems with cognition and mood, and often encounter problems with societal participation [5, 6]. Previous studies in patients with stroke show that cognitive symptoms or changes in social functioning contribute very little to the variance in mRS scores [7]. No studies have yet investigated whether the mRS captures cognitive or neuropsychological complaints in patients with aSAH.

As the mRS is frequently used as a primary endpoint in trials for patients with aSAH, it is important for both researchers and clinicians that its validity is assessed. We previously showed that mRS scores differ significantly when obtained using different assessment methods [8]. In the current study, we will evaluate whether the mRS truly measures global disability in patients with aSAH, including neuropsychological complaints. Furthermore, as recovery after aSAH is a long-term process, the mRS has to be responsive to change in patient condition [9, 10]. The aim of this study is to assess the convergent validity and responsiveness of the mRS in patients with aSAH. Additionally, we aim to compare the distribution patterns between the mRS and patient-reported outcome measures (PROMs), as well as to explore whether various assessment methods of the mRS result in different corresponding PROM scores.

## Methods

### Study design and participants

In this prospective, randomized study, patients were enrolled from six hospitals in the Netherlands between November 2018 and September 2020. The study protocol was registered in the Netherlands Trial Register (NTR number NL7859). This work is part of a randomized controlled trial, in which the inter-method reliability of the mRS was also assessed; therefore the population was necessarily randomized into groups assessed using the mRS obtained by a structured interview or completed through self-assessment (Supplemental Fig. 1). The inclusion criteria were a recent aSAH (≤ 6 weeks ago) and an age ≥ 18 years old. Patients were excluded if they were non-fluent in Dutch or not able to visit the outpatient clinic for follow-up. This study was exempted from ethical approval under Dutch law by the local Medical Ethics Committee, because there was a negligible impact on patients (i.e., completing questionnaires) and treatment remained unchanged. All patients or their representatives gave written informed consent.

### Procedures

The demographic information (age, sex, date of aSAH and date of hospital discharge), World Federation of Neurological Surgeons score (WFNS-score) on admission, modified Fisher score, and location of the aneurysm of each patient were extracted from medical records. Data were collected both at six weeks and six months after discharge. Three assessment methods were used to obtain the mRS: the mRS score was determined by the attending physician (mRS-physician) and subsequently, depending on randomization, by structured interview (mRS-SI) or by self-assessment (mRS-SA). There were no specific guidelines for the assessment by the physician and the assessment could be done face-to-face or by telephone. The Dutch version of the structured mRS interview was used [11]. All assessors of the mRS-SI, were trained by completing an online learning module prior to the start of the study. In the absence of a golden standard for the assessment of the mRS, we considered the mRS-SI to be the best option due to its extensive and structured approach, as well as high inter-rater reliability [12–14]. Therefore, the mRS-SI was used as the main comparator in all analyses. All patients also completed the following PROMs: EuroQoL 5D-5L (EQ-5D-5L) [15], research and development-36 (RAND-36) [16] and the short version of the Stroke Specific Quality of Life scale (SS-QoL) [17]. At the six-month follow-up, patients were also requested to complete the Global Perceived Effect (GPE) [18].

### PROMs for determining convergent validity and responsiveness

The aforementioned PROMs were used as comparator instruments. Multiple PROMs were chosen to cover the mRS construct, because no single PROM measures the same construct as the mRS.

EQ-5D-5L measures general health status and consists of five items: mobility, self-care, daily activities, pain, and anxiety [15]. Each item is scored on a five-point scale. The scores of the EQ-5D-5L items are converted into a total score using the Dutch national value set [19]. EQ-5D-5L was chosen as it is one of the most frequently used questionnaires for measuring general health. It has well-established psychometric properties, including construct validity and responsiveness [20, 21].

RAND-36 is a questionnaire measuring general health status. It includes physical functioning, role limitations due to physical and emotional problems, bodily pain, general health perceptions, vitality, social functioning, and general mental health [22]. The results of RAND-36 can be presented as two summary scores, the physical component summary (PCS) score and the mental component cummary (MCS) score [16]. RAND-36 was chosen because it is one of the most used general health questionnaires, includes more domains than EQ-5D-5 L, and has well-established psychometric properties (except responsiveness) in patients with stroke [23].

SS-QoL is a disease-specific quality of life measure that encompasses 12 domains (social roles, mobility, energy, language, self-care, mood, personality, thinking, upper extremity use, family role, vision, and work/productivity), which can be summarized into a physical and a psychosocial subscore. A short version of SS-QoL was used, which was previously validated in patients with aSAH [17, 24]. SS-QoL was included as it is a disease-specific outcome measure and incorporates items about neuropsychological outcomes in patients with stroke.

GPE was used as an anchor to evaluate the responsiveness of the mRS. It consists of one question about the perceived recovery after the onset of disease. Its response options are on a seven-point scale: very much better, much better, a little better, no change, a little worse, much worse, very much worse [18, 25].

### Convergent validity and responsiveness

The definitions used for convergent validity and responsiveness are based on the consensus on taxonomy, terminology and definitions reached by the Consensus-Based Standards for the Selection of Health Measurement Instruments (COSMIN) panel [26]. Convergent validity is assessed by evaluating the degree to which the scores of a measurement instrument are consistent with the formulated hypotheses, such as the relationship with other instruments. The correlation coefficients of the instrument under study with other instruments are compared to a priori hypotheses about the expected correlations. A positive score for convergent validity is reached when at least 75% of the hypotheses are met [26]. Responsiveness is the ability of an outcome instrument to detect change over time [26]. This can be calculated using an anchor, such as GPE, or by using a different outcome instrument with formulated hypotheses about the expected correlation. A correlation coefficient of 0 to 0.19 was considered very weak, 0.20 to 0.39 weak, 0.40 to 0.59 moderate, 0.60 to 0.79 strong, and 0.80 to 1.0 very strong [27].

Hypotheses for convergent validity between the mRS-SI and the PROMs:

- We expected a moderate to strong negative correlation (-0.4 to -0.8) between the mRS-SI and EQ-5D-5L. EQ-5D-5L measures general health including pain and anxiety, thus it measures a slightly different construct than the mRS.
- We expected a higher correlation of the mRS-SI with the PCS than with the MCS. RAND-36 measures general health and incorporates more neuropsychological domains (especially with the MCS) than the mRS-SI; therefore, we expected that the correlation between the PCS and the mRS would be higher than with the MCS. We expected this to be true for all three assessment methods of the mRS, but with the greatest difference for the mRS-physician. We expected that, due to the non-structured assessment of the physician, there would be less focus on neuropsychological complaints.
- We expected a moderate to strongly negative correlation (-0.4 to -0.8) between the mRS-SI and SS-QoL. SS-QoL is a stroke-specific outcome instrument and incorporates more outcome domains than the construct of the mRS.

Hypotheses for convergent validity of the different assessment methods of the mRS:

- We expected that the correlation between the mRS-SI or mRS-SA and SS-QoL would be higher than the correlation between the mRS-physician and SS-QoL. We expected that the validity would vary between the assessment methods, and we expected that mRS-SA and mRS-SI might reveal more symptoms than mRS-physician.

Hypotheses for responsiveness:

- We expected a mean change score of the mRS between six weeks and six months around − 0.25 to − 0.5. Recovery after aSAH may take several months or even years [6, 10], but we expected to measure some improvement between the assessments.
- We expected that the mean change score of the mRS would show a moderate to strongly negative correlation (–0.5 to − 0.8) with GPE. We expected the mRS and GPE would both be able to detect health changes, but the change might not completely be the same; therefore we did not expect a very strong nor weak correlation.
- We expected that the change score of the mRS-SI would show a moderate to strongly negative correlation (–0.4 to − 0.8) with SS-QoL, RAND-36 and EQ-5D-5L.

### Data analysis

The data were analyzed using IBM SPSS version 25. Missing data were deleted in a pairwise manner. Descriptive statistics were used to describe participant characteristics. Spearman correlations were used to assess the correlation between the mRS (with various assessment methods) and EQ-5D-5 L, RAND-36, and SS-QoL, and to measure the correlation between the change scores of the mRS and EQ-5D-5L, RAND-36, and GPE (non-normally distributed data). Pearson correlations were used for the correlation between the change score of the mRS and SS-QoL. The distribution of EQ-5D-5L, RAND-36, and SS-QoL across the different mRS scores was graphically displayed with boxplots. This allowed us to visualize, compare, and describe the patterns of distribution between the mRS-SI and the PROMs, the differences in PROM scores for various assessment methods of the mRS, and the variability of PROM scores within mRS scores (i.e., theinterquartile range(IQR)). Floor and ceiling effects of the mRS were described and considered present if more than 15% of responses were in extreme lower or upper categories of the scale, respectively [28].

## Results

In total, 150 patients were included in this study (Table 1). One patient was retrospectively diagnosed with non-aneurysmal SAH and excluded, leaving 149 patients with aSAH in the study population. The median mRS-physician score was 1 (IQR = 1.00), while the median mRS-SI and mRS-SA scores were 2 (IQR = 0.50 and IQR = 1.00, respectively) (Supplemental Table 1). The mRS showed no floor or ceiling effects, although it showed a non-normal left-skewed distribution of scores.

**Table 1:** Patient characteristics

		Total (n = 149)	mRS - structured interview (n = 75)	mRS - self-assessment (n = 74)
Age		58^†^ (11.0)	57^†^ (10.5)	59^†^ (11.6)
Sex				
	Male	39 (26.2%)	19 (25.3%)	20 (27.0%)
	Female	110 (73.8%)	56 (74.7%)	54 (73.0%)
Location of aneurysm				
	Anterior circulation	98 (65.8%)	47 (62.7%)	51 (68.9%)
	Posterior circulation	46 (30.9%)	25 (33.3%)	21 (28.4%)
	Unknown	5 (3.4%)	3 (4.0%)	2 (2.7%)
WFNS grade				
	I	75 (50.3%)	43 (57.3%)	32 (43.2%)
	II	29 (19.5%)	13 (17.3%)	16 (21.6%)
	III	10 (6.7%)	5 (6.7%)	5 (6.8%)
	IV	20 (13.4%)	9 (12.0%)	11 (14.9%)
	V	15 (10.1%)	5 (6.7%)	10 (13.5%)
Modified Fisher score				
	0	1 (0.7%	1 (1.3%)	0
	1	13 (8.7%)	6 (8.0%)	7 (9.5%)
	2	22 (14.8%)	11 (14.7%)	11 (14.9%)
	3	46 (30.9%)	22 (29.3%)	24 (32.4%)
	4	63 (42.3%)	31 (41.3%)	32 (43.2%)
	Missing	4 (2.7%)	4 (5.3%)	0

^†^: mean (standard deviation)

Abbreviations:mRS: modified Rankin Scale; WFNS: World Federation of Neurosurgical Societies

### Hypotheses testing for convergent validity

Three of the four hypotheses regarding convergent validity were true in comparison with mRS-SI (Table 2). There was a moderate negative correlation between mRS-SI and EQ-5D-5L (r = − 0.546), and between mRS-SI and RAND-36 PCS (r = − 0.439). There was a strong correlation between mRS-SI and SS-QoL (r = − 0.671). The correlation between mRS-SI and RAND-36 MCS (r = − 0.574) was higher than the correlation between mRS-SI and RAND-36 PCS; therefore, our hypothesis that there would be a higher correlation of the mRS with PCS than with MCS, is true for mRS-physician and mRS-SA, but does not hold for mRS-SI.

**Table 2:** Testing hypothesis of correlation (Spearman, r) between the mRS scores generated using different assessment methods and the different patient-reported outcome measures

	Expected range of correlation	mRS-SI (Spearman correlation)	mRS-physician (Spearman correlation)	mRS-SA (Spearman correlation)
mRS vs. EQ-5D-5L total score	–0.4 to − 0.8	–0.546 (n = 58)	–0.443 (n = 124)	–0.611 (n = 62)
Hypothesis supported		Yes	Yes	Yes
mRS vs. RAND-36 PCS	Higher correlation with PCS than with MCS	–0.439 (n = 60)	–0.465 (n = 128)	–0.599 (n = 64)
mRS vs. RAND-36 MCS		–0.574 (n = 60)	–0.288 (n = 128)	–0.506 (n = 64)
Hypothesis supported		No	Yes	Yes
mRS vs. SS-QoL total score	–0.4 to − 0.8	–0.671 (n = 56)	–0.417 (n = 121)	–0.699 (n = 61)
Hypothesis supported		Yes	Yes	Yes
	Hypotheses for the convergent validity of different assessment methods: The correlation of mRS-SI and mRS-SA with SS-QoL would be higher than the correlation between mRS physician and SS-QoL	Yes	Yes	Yes
Total number of hypotheses supported		3 out of 4	4 out of 4	4 out of 4

Abbreviations: EQ-5D-5L: EuroQoL-5D-5L; MCS: mental component summary score; mRS: modified Rankin Scale; PCS: physical component summary score; RAND-36: research and development-36; SA: self-assessment; SI: structured interview; SS-QoL: Stroke Specific Quality of Life scale

### Responsiveness

The mean change for mRS-SI was − 0.08 (SD 0.915), for mRS-physician was − 0.14 (SD 0.942), and for mRS-SA was − 0.36 (SD 0.923). Changes in the mRS score increase with an increase in self-rated change according to the GPE (Table 3). Because of insufficient numbers of patients reporting ‘a little-’, ‘much-’, and ‘very much deterioration’, these responses were clustered as ‘deterioration’. Nevertheless, in these three categories the number of patients was still relatively low. The correlation between GPE and mRS-SI was 0.245, between GPE and mRS-physician was 0.186, and for mRS-SA was 0.079. There was a weak negative correlation between the change score of the mRS-SI compared with the change scores of the PROMs (Table 4). None of our a priori hypotheses were met.

**Table 3:** Changes in the mRS scores for categories of improvement of GPE determined using different assessment methods between the six-week and six-month follow-up

GPE	mean change (SD)		
	mRS-physician	mRS-SI	mRS-SA
Very much improved	–0.34 (1.136) n = 35	–0.25 (1.209) n = 20	–0.50 (1.019) n = 14
Much improved	–0.18 (0.806) n = 45	–0.24 (0.752) n = 17	–0.50 (0.673) n = 22
A little improved	0.12 (0.781) n = 17	0.38 (0.744) n = 8	0.13 (1.126) n = 8
No change	0.40 (0.548) n = 5	–0.33 (0.577) n = 3	–1.00 (1.414) n = 2
Deterioration	–0.14 (1.027) n = 14	0.33 (0.516) n = 6	–0.43 (0.787) n = 7

The negative values imply that there is a lower mRS score (i.e. a better outcome). Positive values imply that there is a higher mRS score (i.e. worse outcome)

Abbreviations: GPE: global perceived effect; mRS: modified Rankin Scale; SA: self-assessment; SI: structured interview

**Table 4:** Testing the hypothesis of correlation between the mRS-SI and PROM change scores and between GPE and PROM change scores

Measurement	Mean change (SD)	mRS-SI	Expected range in correlation	Hypothesis confirmed
EQ-5D-5L	0.03 (0.155)	–0.359 Spearman Rho (n = 54)	–0.4 to − 0.8	No
RAND-36 PCS	3.4 (8.825)	–0.309 Spearman Rho (n = 56)	–0.4 to − 0.8	No
RAND-36 MCS	2.8 (10.440)	–0.191 Spearman Rho (n = 56)	–0.4 to − 0.8	No
SS-QoL	0.27 (0.596)	–0.395 Pearson (n = 49)	–0.4 to − 0.8	No
GPE	N/A	0.245 Spearman Rho (n = 54)	–0.5 to − 0.8	No

Abbreviations: EQ-5D-5 L: EuroQoL-5D-5L; GPE: global perceived effect; MCS: mental component summary score; mRS: modified Rankin Scale; N/A.: not applicable; PCS: physical component summary score; SA: self-assessment; SI: structured interview; SS-QoL: Stroke Specific Quality of Life scale

### mRS distribution across PROMs

**Fig. 2:**
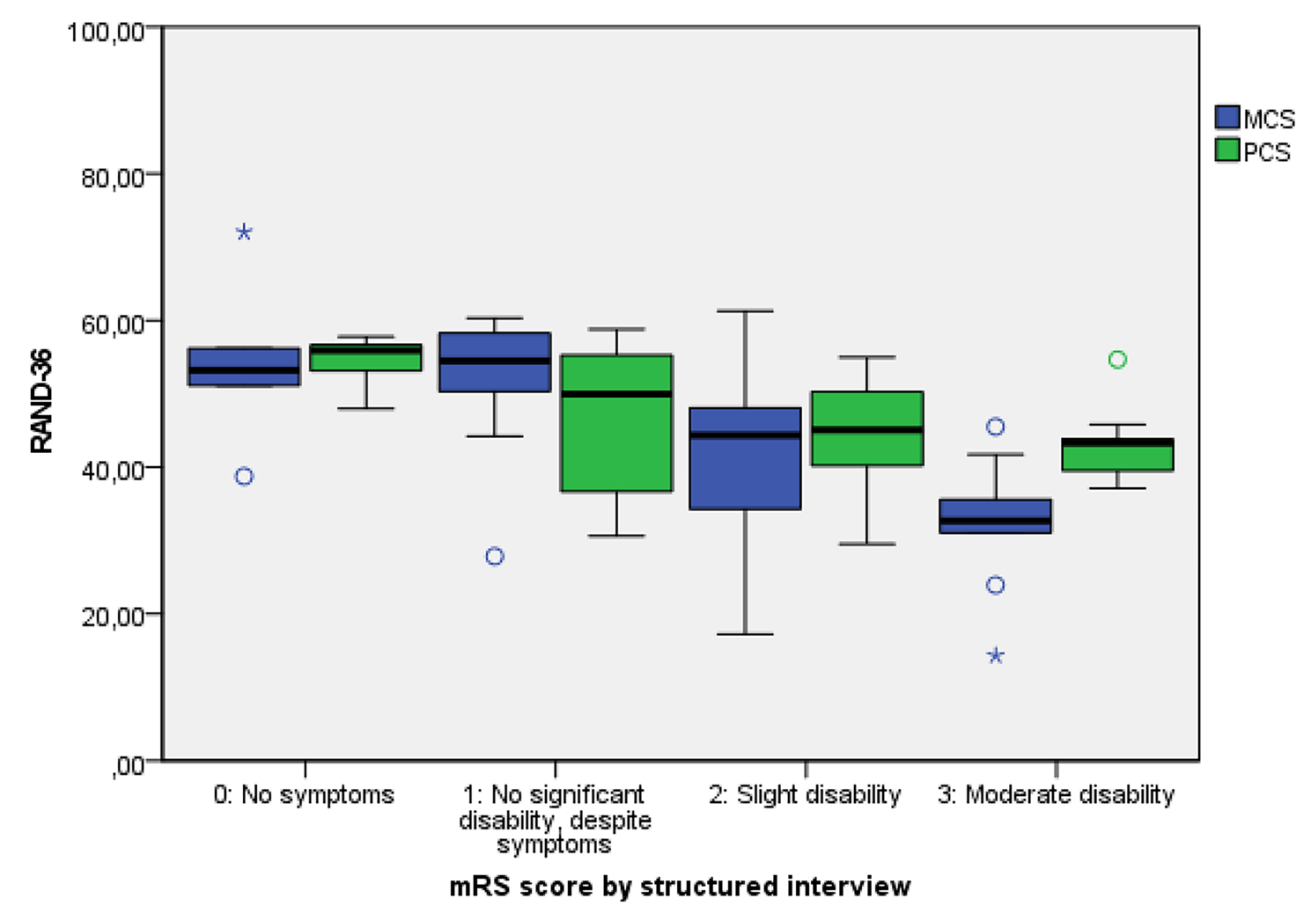
Boxplot of the modified Rankin Scale (mRS) score (0–3) assessed with a structured interview compared to RAND-36 physical component summary (PCS) score and mental component summary (MCS) Note: the thick horizontal bar in the boxes represents the median for each mRS level. The ends of the boxes represent the first and third quartiles. The vertical line represents the minimum and maximum score (value inside 1.5 × interquartile range (IQR)). The open dots represent outliers (outside 1.5 IQR) and the asterisks represent extreme values (outside 3 IQR). Higher mRS scores indicate a worse disability, while higher scores on RAND-36 indicate better function. The RAND-36 scores can range from 0 to 100

The boxplots show to what extent the mRS corresponds with the other questionnaires (Fig. 2, Supplemental Figs. 2 and 3). In general, the higher the mRS score, the lower the median PROM score; however, the median EQ-5D-5L and SS-QoL scores did not differ much between mRS scores of 0 and 1. There was hardly any difference in RAND-36 MCS and SS-QoL total scores for patients with mRS score of 0 or 1. For patients with mRS scores of 1–3, but particularly with an mRS score of 2, the IQRs of the corresponding PROM scores are large. This implies that patients with an mRS score of 2 might have a very high or very low score on the comparator PROM. The discriminant ability of an mRS score of 2 is therefore low.

**Fig. 3:**
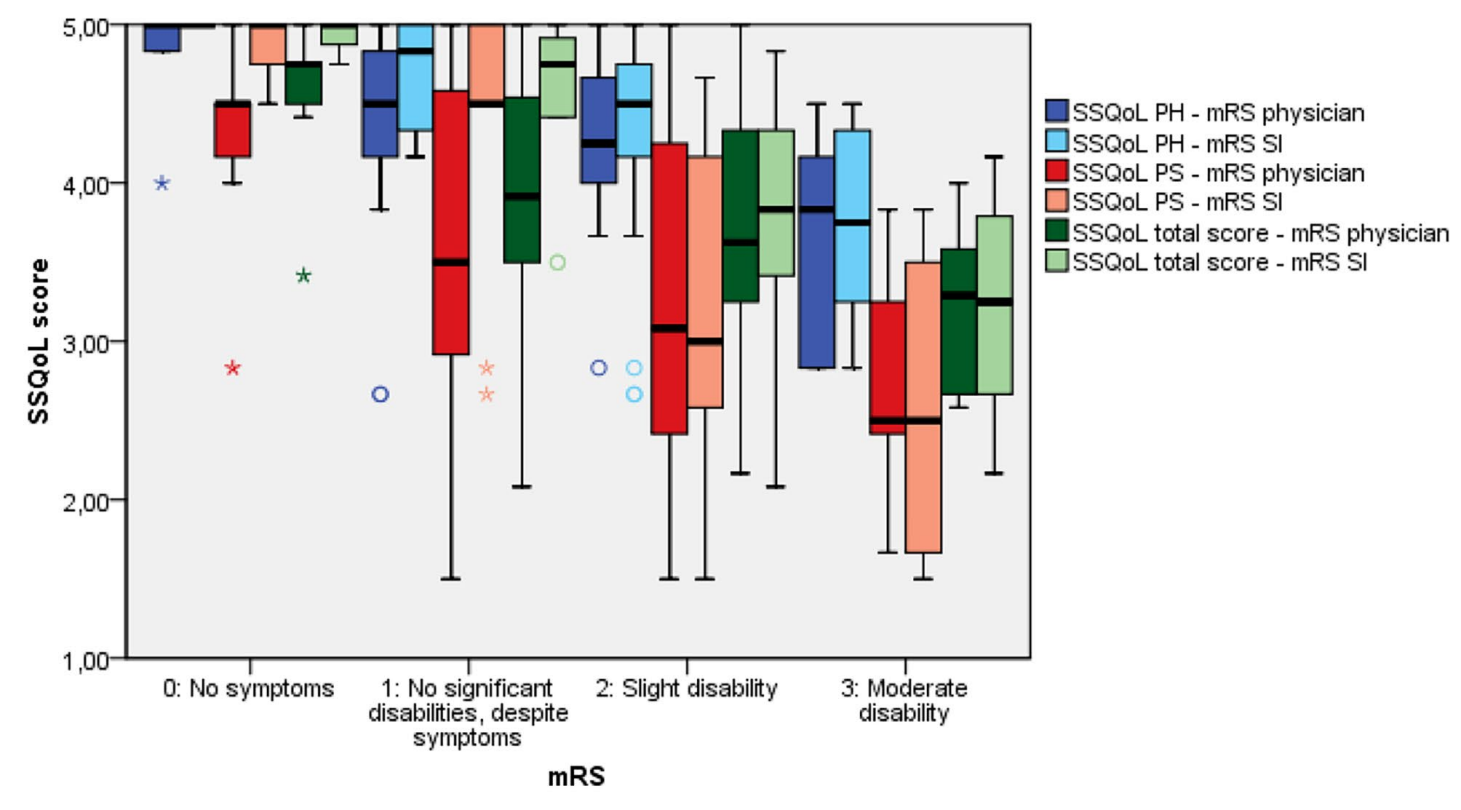
Boxplot of the modified Rankin Scale (mRS) determined using a structured interview (SI) or physician assessment compared to the Stroke-Specific Quality of Life (SS-QoL) scale total score, psychosocial subscore (PS) and physical subscore (PH) six weeks after aSAH Note: the thick horizontal bar in the boxes represents the median for each mRS level. The ends of the boxes represent the first and third quartiles. The vertical line represents the minimum and maximum score (value inside 1.5 × interquartile range (IQR)). The open dots represent outliers (outside 1.5 IQR) and the asterisks represent extreme values (outside 3 IQR). Higher mRS scores indicate a worse disability, while higher scores on SS-QoL indicate better function. The SSQoL scores, both the subscale score and the total score can range from 1 to 5

Figure 3 and Supplemental Fig. 4 show that the variation in the scores between SS-QoL and the mRS is dependent on the mRS assessment method. Patients with an mRS-SI score of 0 or 1 have a higher score (i.e., a better outcome) on SS-QoL psychosocial subscale and a smaller IQR (median = 4.67, IQR = 0.50) than patients with an mRS score of 0 or 1 assessed by a physician (median = 4.33, IQR = 1.50).

## Discussion

This study shows that the mRS generally correlates with other instruments as expected, when applied to patients with aSAH, contributing to evidence towards sufficient convergent validity (75% of hypotheses fulfilled). However, the assessment method of the mRS seems to influence the construct of the mRS, and thus the correlations with other instruments. The correlations of the mRS assessed with various methods and RAND-36 elucidate that disabling neuropsychological complaints are better identified by an mRS assessed using a structured interview than by a physician. The mRS does not seem to be responsive to change between six weeks and six months after aSAH (none of the hypotheses were fulfilled).

### Convergent validity

Although this study supports the sufficient convergent validity of the mRS, some comments can be made. First, the mRS is a global disability scale and measures the construct ‘functional outcome’; [2, 4] therefore, we chose comparator instruments that measure global health outcomes, and not one specific part of the construct of the mRS (e.g., ADL activities). We assigned broad correlation ranges to the hypotheses formulated a priori to measure convergent validity, because it is difficult to provide a more precise indication of the correlation of the mRS with comparator PROMs. They measure a somewhat different construct, and PROMs include neuropsychological complaints that are not specifically assessed by the mRS. This also means that it was relatively easy to fulfill the a priori hypotheses. Second, the correlation coefficient of the mRS with comparator PROMs differs per assessment method. This indicates that, by using different assessment methods to assign an mRS score, different complaints or symptoms are weighted to come to a definitive mRS score. This difference caused a deviation in the number of confirmed hypotheses for validity. Third, pre-existing complaints might have a greater influence on the PROM scores than they have on the mRS score.

### Responsiveness

The mRS does not appear to be sensitive to changes in health outcomes between six weeks and six months after aSAH. Responsiveness has not been thoroughly assessed previously for the mRS. Only one study compared the responsiveness of the mRS in patients after stroke and concluded that the mRS was less sensitive to change than other outcomes [29]. Due to the poor correlation of the mRS with GPE and other PROMs, the question arises of whether GPE, EQ-5D-5L, RAND-36, and SS-QoL are suitable comparator instruments. The responsiveness of the EQ-5D is moderate [21, 30], while for the RAND-36 it is unclear [31, 32], and the SS-QoL is not responsive [33]. In these studies, however, the appropriate methods for assessing responsiveness according to COSMIN criteria are not always used [26]. GPE has proven to be a reliable measure to detect recovery based on ADL limitations, although one can question whether it truly reflects change, or just the current health state [25]. Additionally, GPE measures the change between the health situation directly after aSAH and the health state after six months, while the mean change of the mRS is a measurement of the health change between six weeks and six months after aSAH. The low correlation between the mRS and GPE could therefore be caused by the different time intervals over which the change is measured. This would imply that either (1) the mRS is not sensitive to change, (2) most of the recovery occurs in the first six weeks, or (3) recovery takes place between six weeks and six months, but not in the domains mobility and (I)ADL tasks.

We know that patients with aSAH show improvement of symptoms in the months after aSAH however [6, 10]. The poor responsiveness of the mRS suggests that clinical trials using the mRS may fail to detect a clinically significant difference measured over time. It is also important for clinicians to realize that, due to its poor responsiveness, the mRS is not a suitable instrument to measure improvement in individual patients in clinical practice.

### Neuropsychological complaints

Our study and previous studies illustrate that most patients with aSAH and good functional outcomes —according to the mRS— still suffer from subjective impairments, such as cognitive deficits, depressive symptoms, and anxiety [5, 34]. A structured interview of the mRS appears to detect disabilities caused by neuropsychological outcomes better than a physician’s assessment. If these symptoms are not assessed in detail, the symptoms and their impact might remain undetected and thus not reflected in the mRS score.

Our data show that patients with an mRS-physician score of 0 or 1 have more psychosocial complaints, based on corresponding SS-QoL scores than patients scoring 0 or 1 on mRS-SI assessment. This implies that disabling neuropsychological complaints are better evaluated with a structured interview. Second, the differences in correlation between the various mRS assessment methods and RAND-36 MCS and SS-QoL imply that a structured interview or self-assessment detect more neuropsychological complaints than a physician’s assessment. As patients with an apparently good outcome still have relevant neuropsychological impairments [34–37], it is important to assess neuropsychological outcomes, either with a cognitive test or with PROMs. In studies using the mRS as the only outcome measure, it is important to assess the mRS with a structured interview to better incorporate the neuropsychological outcomes.

### Other considerations in the assessment of the mRS

The mRS is an ordinal scale, with unequal degrees of difference between scores. This makes differentiating between some mRS scores more difficult than between other mRS scores. A low specific agreement for the midrange mRS scores was mentioned earlier [8]. A limitation of the mRS is that a single mRS score can be broadly interpreted. Patients may show an improvement in functioning, such as improving from not being able to work to being able to do 90% of their work, but still have the same mRS score. This on its own has implications for the responsiveness.

Additionally, the IQR of PROM scores per mRS score is lower for an assessment with a structured interview than with a physician’s assessment. This implies that mRS categories are more homogenous if assigned with a structured interview (Fig. 2). This is especially important for the midrange of mRS scores, where the ability of the mRS to discriminate between high or low scores on the comparator PROMs is low.

### Directions for future research and clinical practice

The mRS is considered a preferred measure in core outcome sets for studies in patients with aSAH [38]. Based on our results, it is important to realize that the mRS does not capture all complaints after aSAH, but does measure part of the functional outcome. Furthermore, disabilities caused by neuropsychological complaints are best detected using the mRS-SI. Before the mRS-SA can be used in practice it is important to perform a cognitive validation study. As the mRS shows poor responsiveness, it cannot be used to measure improvement at multiple time points after an intervention.

The question remains how outcomes can be best assessed in patients with aSAH. Currently, no objective outcome measure is available specifically designed for aSAH and without limitations. The Glasgow Outcome Scale Extended has been used in many clinical trials, but shows less discriminative power than the mRS between three months and 12 months after aSAH [38]. Because most therapeutic interventions in aSAH aim to improve neurological deficits and corresponding disability, the use of an additional PROM should be considered in future trials. The available PROMs for use in patients after aSAH were evaluated in a review [39]. Another example is the SOS-SAH [40], a disease-specific PROM that measures often undetected symptoms in patients with aSAH and mild disabilities.

### Limitations

This study has several limitations. First, the sample size for some mRS scores (3,4, or 5) was relatively low. This may limit the generalizability of the results to patients with the worst aSAH outcomes. The potential for patients to show an improvement in complaints might be higher in patients with more complaints and thus a higher mRS score, few of which were included in this study. Therefore, while the responsiveness of the mRS for patients with milder complaints was shown to be insufficient in this study, more research is needed to elucidate the responsiveness of the mRS in the aSAH population as a whole. The study design, with two randomized groups and various assessment methods for the mRS was necessary to evaluate inter-method reliability, but resulted in relatively small patient groups per assessment method when assessing their validity. Furthermore, the limited suitability of the comparator instruments and the different construct of the mRS for its different scores make it hard to formulate well-defined but fair hypotheses for the mRS to test its validity. The results of this study thus provide only a limited contribution of evidence for acceptable convergent validity.

## Conclusions

This study contributes towards the evidence regarding the sufficient convergent validity of the mRS, but shows that it lacks responsiveness. For future studies in patients with aSAH using the mRS as an outcome measure, we advise using a structured interview to assess the mRS rather than a physician’s assessment or self-assessment. Furthermore, we advise against the use of the mRS to measure improvement at multiple timepoints after aSAH.

### Electronic supplementary material

Below is the link to the electronic supplementary material.

**Supplementary Material 1: Supplemental Table 1** Frequency distribution of the modified Rankin Scale (mRS) scores at six weeks after discharge, determined by the physician or based on a structured interview or self-assessment. **Supplemental Figure 1** Study design. **Supplemental Figure 2** Boxplot of the modified Rankin Scale (mRS) score (0?3) assessed with a structured interview compared to EuroQoL-5D-5L (EQ-5D-5L). **Supplemental Figure 3** Boxplot of the modified Rankin Scale (mRS) score (0?3) assessed with a structured interview compared to Stroke Specific Quality of Life (SS-QoL) scale total score and subscores. **Supplemental Figure 4** Boxplot of the modified Rankin Scale (mRS) score (0?3) determined with a self-assessment or physician assessment compared to Stroke Specific Quality of Life (SS-QoL) scale total score, psychosocial subscore (PS), and physical subscore (PH) six weeks after aSAH

## Data Availability

The datasets generated and analyzed during the current study are not publicly available due to the lack of specific patient consent to disclose the data, but are available from the corresponding author upon reasonable request.
