# Supplementary material for: Validity of the modified Rankin Scale in patients with aneurysmal subarachnoid hemorrhage: a randomized study
Source: BMC Neurol. 2024 Jan 12;24:23. doi: 10.1186/s12883-023-03479-x (PMC10785372; doi:10.1186/s12883-023-03479-x)
Supplement: Supplementary file 1 — Supplementary Material 1: Supplemental Table 1 Frequency distribution of the modified Rankin Scale (mRS) scores at six weeks after discharge, determined by the physician or based on a structured interview or self-assessment. Supplemental Figure 1 Study design. Supplemental Figure 2 Boxplot of the modified Rankin Scale (mRS) score (0?3) assessed with a structured interview compared to EuroQoL-5D-5L (EQ-5D-5L). Supplemental Figure 3 Boxplot of the modified Rankin Scale (mRS) score (0?3) assessed with a structured interview compared to Stroke Specific Quality of Life (SS-QoL) scale total score and subscores. Supplemental Figure 4 Boxplot of the modified Rankin Scale (mRS) score (0?3) determined with a self-assessment or physician assessment compared to Stroke Specific Quality of Life (SS-QoL) scale total score, psychosocial subscore (PS), and physical subscore (PH) six weeks after aSAH [file 12883_2023_3479_MOESM1_ESM.docx]

**SUPPLEMENTAL MATERIAL**

Supplemental Table 1
Frequency distribution of the modified Rankin Scale (mRS) scores at six weeks after discharge, determined by the physician or based on a structured interview or self-assessment.

|  | **Randomized to mRS structured interview (n = 59)** | |  | **Randomized to mRS self-assessment (n = 56)** | |
| --- | --- | --- | --- | --- | --- |
| **mRS** | **Physician assessment** | **Structured interview** |  | **Physician assessment** | **Self-assessment** |
| 0 (no symptoms) | 8 (13.6%) | 5 (8.5%) |  | 7 (12.5%) | 8 (14.3%) |
| 1 (no significant disability, despite symptoms) | 23 (39.0%) | 10 (16.9%) |  | 24 (42.9%) | 1 (1.8%) |
| 2 (slight disability) | 19 (32.2%) | 31 (52.5%) |  | 11 (19.6%) | 25 (44.6%) |
| 3 (moderate disability) | 6 (10.2%) | 10 (16.9%) |  | 8 (14.3%) | 14 (25.0%) |
| 4 (moderately severe disability) | 2 (3.4%) | 2 (3.4%) |  | 3 (5.4%) | 0 |
| 5 (severe disability) | 1 (1.7%) | 1 (1.7%) |  | 3 (5.4%) | 8 (14.3%) |

Patients that were missing either a physician mRS score or an mRS score from either the structured interview or the self-assessment were excluded from the analysis. Patients that had the structured interview or self-assessment more than 14 days after the assessment of the physician were excluded, as it could not be guaranteed that a difference in mRS score was caused by a difference in scoring rather than by a change in health status.

Supplemental Figure 1
Study design

Abbreviations: aSAH: aneurysmal subarachnoid hemorrhage; EQ-5D-5L: EuroQoL-5D-5L; GPE: global perceived effect; mRS: modified Rankin Scale; RAND-36: research and development-36; SS-QoL: Stroke Specific Quality of Life scale.

Supplemental Figure 2
Boxplot of the modified Rankin Scale (mRS) score (0–3) assessed with a structured interview compared to EuroQoL-5D-5L (EQ-5D-5L).


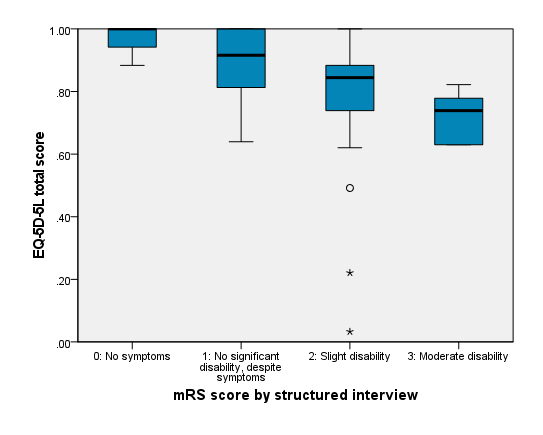


Note: the thick horizontal bar in the boxes represents the median for each mRS level. The ends of the boxes represent the first and third quartiles. The vertical line represents the minimum and maximum score (value inside 1.5 × interquartile range (IQR)). The open dots represent outliers (outside 1.5 IQR) and the asterisks represent extreme values (outside 3 IQR). Higher mRS scores indicate a worse disability, while higher scores on the EQ-5D-5L indicate better function. mRS scores 4 and 5 were removed from the figure due to the few number of cases. The EQ-5D total score can range from 0 to 1.

Supplemental Figure 3
Boxplot of the modified Rankin Scale (mRS) score (0–3) assessed with a structured interview compared to Stroke Specific Quality of Life (SS-QoL) scale total score and subscores.


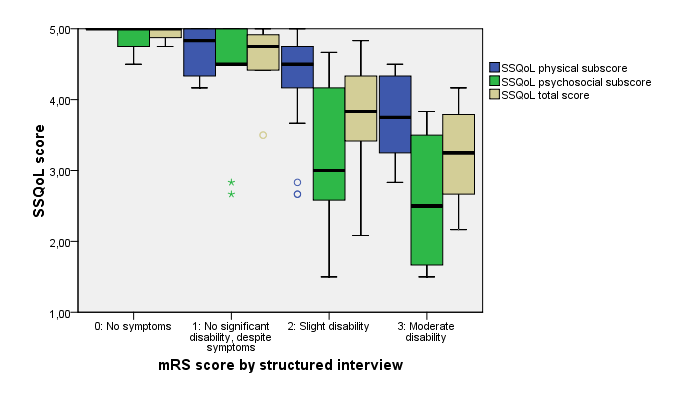


Note: the thick horizontal bar in the boxes represents the median for each mRS level. The ends of the boxes represent the first and third quartiles. The vertical line represents the minimum and maximum score (value inside 1.5 × interquartile range (IQR)). The open dots represent outliers (outside 1.5 IQR) and the asterisks represent extreme values (outside 3 IQR). Higher mRS scores indicate a worse disability, while higher scores on the SS-QoL indicate better function. mRS scores 4 and 5 were removed from the figure due to the few number of cases. Both the SSQoL total score and the subscale score can range from 1 to 5.

Supplemental Figure 4
Boxplot of the modified Rankin Scale (mRS) score (0–3) determined with a self-assessment or physician assessment compared to Stroke Specific Quality of Life (SS-QoL) scale total score, psychosocial subscore (PS), and physical subscore (PH) six weeks after aSAH


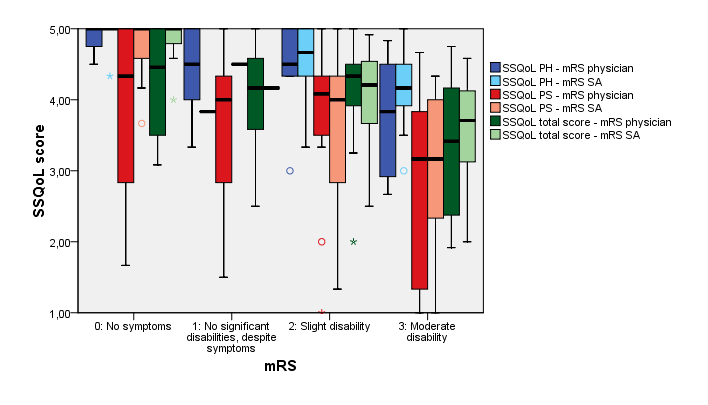


Note: the thick horizontal bar in the boxes represents the median for each mRS level. The ends of the boxes represent the first and third quartiles. The vertical line represents the minimum and maximum score (value inside 1.5 × interquartile range (IQR)). The open dots represent outliers (outside 1.5 IQR) and the asterisks represent extreme values (outside 3 IQR). Higher mRS scores indicate a worse disability, while higher scores on the SS-QoL indicate better function. mRS scores 4 and 5 were removed from the figure due to the few number of cases. Both the SSQoL total score and the subscale score can range from 1 to 5.
